# Consumption

**Published:** 1897-02

**Authors:** 


### Consumption.

The colder the atmospheric air the patient breathes the better; the more oxygen it contains, bulk for bulk, the more it acts as an antiseptic; the more it expands when it has been inspired, and, in expanding, dilates the air cells, the more it tends to cool the overheated lung tissues, rendering them less favorable for the multiplication of bacilli.—*Playter.*

